# O2.6. SOCIAL SIMULATION IN VIRTUAL REALITY IMPROVES EMBODIMENT OF EMOTIONS IN SCHIZOPHRENIA

**DOI:** 10.1093/schbul/sby015.196

**Published:** 2018-04-01

**Authors:** Lénie Torregrossa, Matthew Snodgress, Laura Hieber Adery, Megan Ichinose, Joshua Wade, Dayi BIan, Lauri Nummenmaa, Eric Granholm, Nilanjan Sarkar, Sohee Park

**Affiliations:** 1 Vanderbilt University; 2 Aalto University, University of Turku; 3 UCSD

## Abstract

**Background:**

Bodily-self disturbances and anomalous emotional functioning are core features of schizophrenia that play a major role in social and functional outcome. Much has been written about abnormal perception and expression of emotions in schizophrenia but less is known about the bodily experience of emotions in this population. The prevalence of anomalous bodily-self experiences (Parnas & Handset, 2003), impaired simulation (Park et al, 2008) and interoception deficits (Ardizzi et al., 2016) in schizophrenia suggests that embodiment of emotions might be altered in this population.

**Methods:**

We investigated emotional embodiment in individuals with schizophrenia (SZ) and demographically-matched controls to determine whether SZ experience anomalous bodily sensations of emotions. We then implemented a novel Virtual Reality (VR) social skills intervention that required participants to simulate social interactions with avatars. The VR training was designed to target social attention and improve simulation of other people’s emotions, intentions, and actions in SZ, thereby improving embodiment of emotions.

We recruited twenty-six individuals with schizophrenia (SZ) and 26 demographically matched controls (CO). At baseline, we assessed social functioning, cognitive functions, symptom and embodied emotions. An online body mapping task (Nummenmaa et al., 2014) was used to generate spatial maps of bodily sensations experienced during 14 emotions categories. Then, SZ participated in the 5-week, novel VR social skills intervention that targeted social attention and simulation. Naturalistic scenarios, in which subjects moved through variable sequences of steps to attain the goal of a “mission” (e.g. find out the birthday of the avatar etc.). Subject interacted with an avatar and practiced perspective-taking and pragmatics to advance to the next level of difficulty.

**Results:**

At baseline, bodily sensation maps show overall reduced embodiment of emotions in SZ as compared to CO. Statistical pattern recognition with Linear Discriminant Analysis (LDA) revealed less unique bodily sensations of emotions in SZ. Similarity scores between the maps of CO and SZ revealed a specific deficit in embodiment of low-arousal emotions (i.e. depression, sadness, shame) in SZ. After five weeks of VR training, negative symptoms and emotional embodiment improved in SZ. Specifically, embodiment of low-arousal emotions increased. Moreover, changes in the body maps of emotion indicated increased concordance among SZ.

**Discussion:**

Anomalous embodiment of emotions plays an important role in the poor social outcome of individuals with schizophrenia, but a 5-week VR training of social attention, simulation, perspective taking, and communication skills was effective in improving emotional embodiment. Further research is warranted to elucidate underlying social cognitive mechanisms that link self-disturbances and embodiment of emotions.

